# Therapist's Gender and Gender Roles: Impact on Attitudes toward Clients in Substance Abuse Treatment

**DOI:** 10.1155/2013/591521

**Published:** 2012-12-11

**Authors:** Tytti Artkoski, Pekka Saarnio

**Affiliations:** School of Social Sciences and Humanities, 33014 University of Tampere, Finland

## Abstract

The purpose of the present study was to investigate the impact of therapist's gender and gender roles on attitudes toward clients. Attitudes toward motivational interviewing were also a focus as MI can be hypothesized to be feminine rather than masculine in nature. The subjects (*N* = 246) were Finnish substance abuse therapists. Their attitudes toward clients were measured using a vignette task. Results indicated that female therapists were significantly more positive toward clients than were male therapists. Although females were significantly more feminine than males, they saw themselves as masculine as the males did. The more feminine the therapist was, the more s/he preferred MI. In the future, an examination of this kind should be combined with measurement of treatment processes and outcomes.

## 1. Introduction

The effects of various treatments on outcome have been studied extensively both in psychotherapy and substance abuse treatment [1, 2]. The investigation of between-therapist variation in outcome has been infrequent, although it has proved an important factor in both disciplines [3, 4].

Other little-studied factors are the gender and gender roles of the therapist and their impact on treatment effectiveness [5]. Gender role is a key concept in our study. It refers to the set of attitudes and behaviors socially expected of the members of a particular gender [6]. According to Bem's [7] theory, a traditionally gender-typed person is highly attuned to the cultural definitions of gender-appropriate behavior and uses such definitions as the ideal standard against which her or his own behavior is to be evaluated. Masculinity and femininity are gender roles of the traditional type. Androgyny, in turn, is considered to be a modern gender role. It means that a person is both masculine and feminine; these traits are not mutually exclusive.

Research on psychotherapy has indicated that therapy effectiveness may be predicted on the basis of gendered factors [8]. In the substance abuse field, there is evidence that therapist's attitudes toward clients vary according to the client's gender. DeJong et al. [9] demonstrated that therapists were more confrontational and critical with male clients, while female clients received more empathy and support. The male clients were seen by the therapists as threatening, in which case the attitudes became confrontational, while female clients were seen as submissive, which led to empathetic attitudes. These attitudes were due to stereotypical gender roles common in society. The therapist's own gender had no impact on the attitudes toward clients in the study by DeJong et al.

By contrast, findings on mental health professionals have indicated that males generate more stereotypical attitudes toward clients than do females [10, 11]. These findings corroborate a study by Bernstein and Lecomte [12] reporting that psychotherapist's gender is an important factor for attitudes toward clients, males being more stereotypical than females.

A small study by Saarnio et al. [13] showed that the clients of male therapists dropped out of inpatient substance abuse treatment significantly more frequently than did the clients of female therapists (20 versus 10%). Five therapists of each gender and 105 clients took part. Unfortunately, a more detailed examination of the findings was not possible because no in-session data were collected.

However, one possible explanation is that female therapists were more adept at avoiding alliance ruptures that easily lead to dropping out. This explanation is supported by a recent Finnish study which found that female therapists in substance abuse treatment were significantly more empathetic and friendly toward clients than were their male colleagues [14]. Moreover, avoidance of excessive directiveness was considered more important by female therapists than by males.

There is evidence that therapist empathy is an essential factor in substance abuse treatment [15]. Empathy is significant for the working alliance and thus for the continuity of treatment [16]. Miller et al. [17] demonstrated that therapist empathy explained as much as 67% of variance in treatment outcome; in other words, the more empathetic the therapist, the better the outcome.

Miller et al. [18] showed experimentally that the therapist's style affects the client's drinking after treatment. From among two experimental groups, the therapists in one group were instructed to work in a directive-confrontational style and the therapists in the other group in a client-centered style. The therapists in the second group followed the principles of the motivational interviewing (MI) in which emphasis is placed on a positive attitude toward the client, especially empathy [19]. The directive-confrontational style caused significantly more opposition than did the client-centered style. The treatment results can be summed up as follows: the more the therapist confronted, the more the client drank one year after the treatment.

MI can be hypothesized to be feminine rather than masculine in nature as in it avoidance of confrontation and excessive directiveness play important roles. It would be interesting to find out whether there are between-therapist differences in attitudes toward MI due to gender or gender roles. The hypothesis is wholly explorative in nature.

The purpose of the present study was to investigate the impact of therapist's gender and gender roles on attitudes toward clients with different genders and sexual orientations. Attitudes toward MI were also a focus. These were formulated as three questions:

1. are there differences in masculinity, femininity, or androgyny between male and female therapists?
2. are there between-therapist differences due to gender or gender roles in attitudes toward clients?
3. are gender or gender roles connected with attitudes toward MI?

## 2. Method

### 2.1. Subjects

The subjects (*N* = 246) were Finnish substance abuse therapists employed by the A-Clinic Foundation. The A-Clinic Foundation has treated clients with various addictions since 1955, and today it provides about 40% of the substance abuse treatment in Finland.

An electronic questionnaire was sent via the Internet to counselors, social workers, nurses, physicians, psychologists, and team leaders (*N* = 546). Regardless of job title, they all had the same task, therapy with clients. Therefore, for simplicity, they are called as therapists. The response rate was 45.1%. Unfortunately, we did not get any information on nonrespondents. This was due to an anonymous procedure in data collection.

Out of those participating in the study, 29.7% were men (*n* = 73) and 70.3% women (*n* = 173). The gender distribution was similar to that of the total personnel of the A-Clinic Foundation (22.2% men and 77.8% women). The age of the subjects varied between 24 and 63 years (M = 44.0; SD = 10.0), while the women (M = 42.8; SD = 9.9) were significantly (*t*_244_ = 3.3; *P* = 0.001) younger than the men (M = 47.2; SD = 9.5).

On average, the female therapists had a higher level of professional education than the male therapists, even though the males had acquired university degrees more often than their female colleagues (Table 1). Among therapists in team leader position, men were more common than women. Compared to men, the women more often worked as nurses. Every tenth subject had a history of personal recovery of substance abuse.

Male therapists reported more often being homosexual than did female therapists. According to a population-based Finnish study, the proportion of individuals with gay, lesbian, or bisexual identities has been estimated to be 2.5% of males and 1.9% of females in the year 2007 [20].

### 2.2. Materials

The first part of the questionnaire contained 18 items eliciting background information. The question on MI was formulated as follows: “In motivational interviewing one avoids directly telling the client what to do. How important do you consider this principle to be in substance abuse treatment?” The subjects were requested to use a five-point scale (1 =  not so important*⋯*5 =  very important).

The different gender roles of the subjects, which in this study included masculinity, femininity, and androgyny, were measured by the Bem Sex-Role Inventory (BSRI) [21]. For this purpose, a short version of the BSRI consisting of 30 items was translated into Finnish by an expert translator. Backtranslation was not used as there were only single adjectives to translate.

Each item, such as “I am gentle” or “I am assertive,” was rated on a seven-point scale (1 = never or almost never true*⋯*7 = almost or almost always true). Both masculine and feminine traits were measured by ten items. In addition, ten neutral fillers were included.

The androgyny score is the difference between an individual's femininity and masculinity. A high positive score indicates femininity and a high negative score indicates masculinity; the closer the score is to zero, the more androgynous the person is.

The alpha reliabilities of masculinity and femininity in the present study were of the same level as in the original study [7]: 0.78 (0.86) and 0.81 (0.81). It was not possible to calculate the alpha reliability for androgyny.

Despite having been developed over 30 years ago, the BSRI continues to be extensively used in both research and clinical work. In addition, its psychometric properties are considered valid [22]. However, recent studies have reanalyzed and questioned the BSRI's factor structure and validity [23, 24].

The attitudes of the therapists toward clients were measured with a vignette task, in which the subject had to associate adjectives with three different fictional client cases. The cases differed from each other only as regards the client's gender and sexual orientation. The first case was a heterosexual male with a substance use disorder. The second vignette had to do with a heterosexual female, and the third vignette concerned a homosexual male.

The vignette rating made use of 50 adjectives extracted from the Adjective Check List (ACL), which is commonly used to measure personality traits [25]. Half of adjectives were negative and half positive in meaning. The subjects selected six adjectives for each vignette.

Like the BSRI, the ACL has a wide range of uses and applications in both research and clinical work. In addition, the validity of the ACL has been found to be high [25].

### 2.3. Procedure

The study was approved by the A-Clinic Foundation's Ethics Committee. The electronic questionnaire was sent to the therapists via the Internet. The participation of the therapists was voluntary and anonymous.

 The statistical analyses were carried out using SPSS software (version 16.0). The *χ* ^2^ test, *t*-test, correlations, and analyses of variance (ANOVA, repeated measures, and MANOVA) were used. Effect sizes were calculated with Cohen's *d*, defined as the difference between the means of female and male therapists, divided by the pooled standard deviation of these groups.

## 3. Results

### 3.1. Gender and Gender Roles

First, the results on gender roles measured by the BSRI were compared between male and female therapists. The raw scores were not converted to *t*-scores because they were based on normative data over 30 years old. In addition, the results were not compared to those of other populations.

The male therapists received a slightly higher mean score for masculinity (M = 4.9; SD = 0.6) than the female therapists (M = 4.8; SD = 0.6). According to the *t*-test, the difference was not statistically significant (*t*_244_ = 0.7; *P* = 0.5). Effect size was small (*d* = 0.1).

As for femininity, the means between males (M = 5.2; SD = 0.6) and females (M = 5.5; SD = 0.6) differed significantly (*t*_244_ = 2.6; *P* = 0.01) from each other. The male therapists were less feminine than the females. However, effect size was small (*d* = 0.3).

As regards the androgynous gender role, the male (M = 0.3; SD = 0.8) and female therapists (M = 0.6; SD = 0.8) differed significantly (*t*_244_ = 2.5; *P* = 0.01) from each other. Effect size was small (*d* = 0.3). The mean score of female therapists deviated more from zero than that of men, so the male therapists were more androgynous than the women.

### 3.2. Attitudes toward Clients

**Figure 1:**
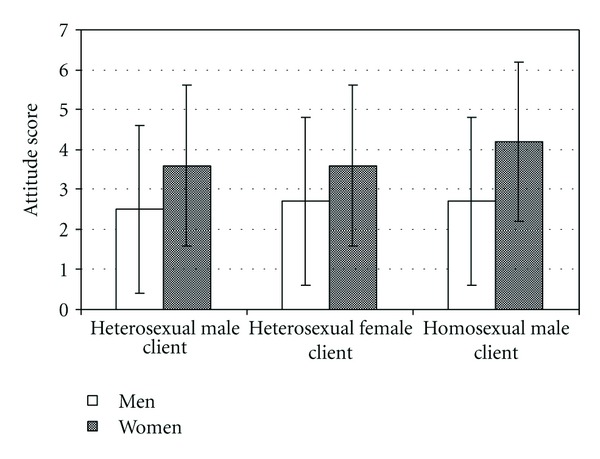
Means and standard deviations (±) for men (*n* = 73) and women (*n* = 173) in attitudes toward clients as measured by the vignette task. Total number of positive adjectives was used as an attitude score.

In the next section of the questionnaire, the therapists had to select six adjectives that best described the client in each of the three vignettes. Gender roles were not significantly connected with the ratings. Instead, gender as such was a significant factor: female therapists had a more positive attitude than the males toward all cases (Figure 1). A repeated analysis of variance indicated that the difference between the columns was significant (*F*_1,244_ = 19.8; *P* = 0.000).

On the basis of the *t*-test, the difference between genders was greatest in attitudes toward the homosexual male client (*t*_244_ = 5.4; *P* = 0.000) and smallest in attitudes toward the heterosexual female client (*t*_244_ = 3.1; *P* = 0.002). The genders also differed significantly (*t*_244_ = 4.0; *P* = 0.000) from each other in their attitudes toward the heterosexual male client. Effect sizes for vignettes were medium to large (*d* = 0.8; 0.6; 1.1).

When controlling for background variables described in Table 1, the one-way analysis of variance revealed three variables on the basis of which the therapists differed significantly from each other as regards their attitudes toward the vignettes: professional education, job title, and technical eclecticism. When these variables and gender were used as independent variables and the combined score of the vignettes as a dependent variable in MANOVA, only technical eclecticism (*F*_3,235_ = 3.1; *P* = 0.03) and gender (*F*_3,235_ = 7.4; *P* = 0.000) remained significant. Eclectic therapists were more positive toward vignettes when compared with single-method therapists.

In addition, ANOVA indicated that the therapist's marital status and length of therapy training interacted significantly with gender on the attitudes toward the vignettes (Table 2). Single men had more positive attitudes toward all vignettes than men in pair relationships. For women the inverse was true: female therapists in a pair relationship had a more positive attitude than single women toward all cases. As for lengthy therapy training, the attitudes of the male therapists who had completed lengthy training were more positive, while among the female therapists such training weakened the positive client images.

### 3.3. Attitudes toward MI

The study also focused on the connections between gender, gender roles, and attitude toward MI. Femininity had a significant positive correlation (*r* = 0.18; *P* < 0.01) with attitude toward MI: as the therapist's femininity increased, so did the preference for MI. Masculinity and androgyny did not correlate significantly with the attitude score.

MI was slightly more important for female therapists (M = 4.0; SD = 0.7) than in males (M = 3.8; SD = 0.7). However, they did not differ significantly (*t*_244_ = 1.3; *P* = 0.2) from each other. Effect size was small (*d* = 0.2). When controlled for, MI usage per se was not significantly connected with preference for MI.

## 4. Discussion

The present study investigated the impact of therapist's gender and gender roles on attitudes toward clients. Attitudes toward motivational interviewing were also a focus. 

On average, the female therapists were significantly more feminine than the male therapists. However, masculinity was at the same level in both genders. The degree of androgyny was higher among the male therapists than among the females. 

Female therapists were significantly more positive than male therapists in their attitudes toward all the cases in the vignette task. This finding indirectly supports Saarnio's [14] conclusion: “On the grounds of personality and interpersonal functioning, female therapists were keener on working according to motivational interviewing” (page 1470).

Our finding differs from the conclusion of DeJong et al. [9] that therapist's gender has no impact on attitudes toward clients. On the contrary, the vignette task result corroborates the findings for mental health professionals, indicating between-gender differences in attitudes toward clients [10–12].

Client's gender was not a relevant factor for therapist's attitudes: the vignette task scores were at the same level for both male and female cases. This finding also differs from the result of DeJong et al. [9] that depending on the client's gender, substance abuse therapists will prefer different attitudinal stances.

As to why the female therapists had the most positive attitudes toward the homosexual male client, no answer can be given on the basis of the present study. However, in an international comparison, there were population-level differences between the genders in attitudes toward homosexuals, particularly in the Nordic countries [26]. Women were more positive toward homosexuals.

The topic is an important one, since some studies have indicated that homosexuals may have a higher risk of substance use disorders than heterosexuals [27]. In addition, it has been demonstrated that homosexuals have difficulties in entering substance abuse services [28]. These findings could be taken into account in therapist training, especially for men.

We hypothesized MI to be feminine rather than masculine in nature as in it avoidance of confrontation and excessive directiveness play important roles. The results indicated that gender as such had no significant association with the therapist's attitude toward MI, while a significant correlation was found between femininity and the attitude score: the more feminine therapist was, the more s/he preferred MI. To the best of our knowledge, there are no studies available on this subject.

Our findings showed that eclectic therapists were more positive toward clients when compared with single-method therapists. This may be considered as an indication of more expertise among the eclectic therapists. We also found that therapist's marital status and length of therapy training interacted significantly with gender on attitudes toward the vignettes. However, these interactions were explorative in nature, and no parallel information was available. It is therefore difficult to discuss them without the risk of speculation. A more detailed analysis of this topic is desirable. 

 There were certain limitations in the present study. One of them was the modest response rate. Although the questionnaires sent via the Internet had a high degree of anonymity, more responses might have been obtained if data had been gathered through personal contacts. Second, while the BSRI has been used extensively, there are questions and limitations as to how valid its underlying factors currently are [23, 24]. Third, the assessment of attitudes toward clients could partly have been confounded by socially desirable responding [29]. Tourangeau and Yan [30] concluded in their review that responses to questions on sexual orientation, in particular, are often based on social expectations. Fourth, attitudes toward MI were only measured by a single item. Consequently, the measurement was not optimally reliable.

 What the findings mean for practical substance abuse treatment remains open. In the future, an examination of this kind should be combined with the measurement of treatment processes and outcomes. Are there differences between female and male therapists in the continuity and outcome of substance abuse treatment? What do the gender roles matter for everyday treatment practice? These are necessary steps before we can draw sound conclusions on gendered effects in this field.

## Figures and Tables

**Table 1:** Background information on therapists by gender. Comparison used *χ* ^2^ test.

	Men (*n* = 73)		Women (*n* = 173)		*P*
	*n*	%	*n*	%	
Marital status					ns
Single	13	17.8	22	12.7	
Cohabiting	15	20.5	46	26.6	
Married	38	52.2	80	46.3	
Divorced or separated	5	6.8	21	12.1	
Widowed	2	2.7	4	2.3	
Sexual orientation					0.01
Heterosexual	66	90.5	167	96.5	
Bisexual	2	2.7	5	2.9	
Homosexual	5	6.8	1	0.6	
Professional education					0.001
Brief professional education	2	2.7	1	0.6	
School level	16	21.9	16	9.2	
College or polytechnic	31	42.5	119	68.8	
University	24	32.9	37	21.4	
Job title					0.008
Counselor	25	34.2	52	30.1	
Social worker	11	15.1	33	19.1	
Nurse	6	8.2	44	25.4	
Physician or psychologist	7	9.6	7	4.0	
Team leader	24	32.9	37	21.4	
Experience in substance abuse treatment					0.05
Under 5 years	14	19.2	59	34.1	
5–15 years	33	45.2	71	41.0	
Over 15 years	26	35.6	43	24.9	
Technical orientation					ns
Cognitive therapies	9	12.3	19	11.0	
Motivational interviewing	4	5.5	11	6.3	
Solution-focused	15	20.5	27	15.6	
System theoretical	3	4.1	2	1.2	
Psychodynamic	2	2.7	6	3.4	
Community treatment	14	19.2	22	12.7	
Eclectic	22	30.2	84	48.6	
None of these	4	5.5	2	1.2	
Lengthy therapy training*					ns
Yes	25	34.2	53	30.6	
No	48	65.8	120	69.4	
Personal recovery of substance abuse					0.001
Yes	14	19.2	10	5.8	
No	59	80.8	163	94.2	

*At least two years of intensive therapy training.

**Table 2:** Interactions of gender with marital status and length of therapy training on attitudes toward cases in the vignette task (*N* = 246). Analysis used ANOVA.

	*F*	Df	*P*
Heterosexual male client			
Gender × marital status	6.9	1,242	0.009
Gender × length of therapy training	2.0	1,242	0.16
Heterosexual female client			
Gender × marital status	4.8	1,242	0.03
Gender × length of therapy training	6.4	1,242	0.01
Homosexual male client			
Gender × marital status	5.8	1,242	0.02
Gender × length of therapy training	5.2	1,242	0.02
